# Specialization of the brain for language in children with Fragile X Syndrome: a functional Near Infrared Spectroscopy study

**DOI:** 10.1186/s11689-024-09582-5

**Published:** 2024-12-19

**Authors:** Elizabeth Smith, Kelli C. Dominick, Lauren M. Schmitt, Ernest V. Pedapati, Craig A. Erickson

**Affiliations:** 1 Division of Behavioral Medicine and Clinical Psychology, Cincinnati Children’s Hospital Medical Center, Cincinnati, OH USA; 2 Department of Pediatrics, College of Medicine, University of Cincinnati, Cincinnati, OH USA; 3 Department of Psychiatry and Behavioral Neuroscience, College of Medicine, University of Cincinnati, Cincinnati, OH USA; 4 Division of Child and Adolescent Psychiatry, Cincinnati Children’s Hospital Medical Center, Cincinnati, OH USA

**Keywords:** Fragile X syndrome, Functional near infrared spectroscopy, Speech perception, Auditory cortex

## Abstract

Specialization of the brain for language is early emerging and essential for language learning in young children. Fragile X Syndrome (FXS) is a neurogenetic disorder marked by high rates of delays in both expressive and receptive language, but neural activation patterns during speech and language processing are unknown. We report results of a functional Near Infrared Spectroscopy (fNIRS) study of responses to speech and nonspeech sounds in the auditory cortex in a sample of 2- to 10-year-old children with FXS and typically developing controls (FXS *n* = 23, TDC *n* = 15, mean age = 6.44 and 7.07 years, respectively). Specifically, we measured changes in oxygenated and deoxygenated hemoglobin in the auditory cortex during blocks of speech and nonspeech matched noise in children with FXS and sex-and-age-matched controls. Similar to controls, children with FXS showed hemodynamic change consistent with neural activation of the primary auditory regions for speech as well as leftward lateralization for speech sound processing, strength of which was associated with higher verbal abilities in FXS. However, while controls showed neural differentiation of speech and nonspeech in the left auditory cortex, children with FXS did not demonstrate differentiation of the two conditions in this study. In addition, the children with FXS showed a greater neural activation to the nonspeech condition overall. Overall, these results suggest that basic patterns of neural activation for speech are present in FXS in childhood, but neural response to nonspeech sounds may differ in FXS when compared to controls.

**Supplementary Information:**

The online version contains supplementary material available at 10.1186/s11689-024-09582-5.

## Introduction

Fragile X Syndrome (FXS), which is the leading genetic cause of both autism spectrum disorder and intellectual disability, is almost universally associated with some degree of language delay and/or impairment [1]. The disorder is most often caused by a CGG triplet repeat expansion in the promotor region of the *FMR1* gene located on the long arm of the X chromosome. This expansion results in gene methylation, inactivation, and decrease or loss of Fragile X Messenger Ribonucleoprotein (FMRP) expression. The process by which variation in FMRP leads to language impairments as well as other neurobehavioral sequelae has been investigated via translational models, with the FXS mouse model supporting pervasive differences in auditory processing related to FMRP loss. These differences in the auditory system are present early in development in the mouse model of FXS [2], indicating potential for cascading effects on the developing brain. Whereas differences in basic auditory processing have also been documented in humans with FXS [3], there is a lack of research on how this relates to neural responses to speech and language stimuli in FXS. Investigating neural responses to speech and nonspeech sounds, especially during childhood in humans with FXS, is necessary to understand how underlying genetic differences drive impairments in language and communication throughout the lifetime.

Based on mouse models, loss of FMRP is associated with global changes in synapse development and maintenance [4] as well as dendritic abnormalities [5]. The specific effects of FMRP on the auditory system have been a particular focus, given that FMRP is expressed in multiple cortical and subcortical regions critical for auditory function [3, 6]. *Fmr1* knockout mice show atypical auditory brainstem responses (ABR’s) compared to wild type mice [6] as well as a heightened response to sounds in cortical neurons [7]. The presence of atypical ABR’s in the mouse model of FXS supports a specific role for FMRP loss in auditory processes in addition to a more general effect across cognitive abilities.

Human studies in adolescents and adults with FXS also show a consistent pattern of atypical auditory processing in FXS. Specifically, adolescents and adults with FXS show heightened neural responses to changes in sounds [8–11] as well as reduced neural habituation to sounds [12]. However, most studies of auditory processing in humans with FXS focus on non-social, nonspeech sounds, making it difficult to determine if and how auditory processing differences in FXS are related to early-emerging and persistent language delays. Only one study to date has investigated neural response to speech sounds in humans with FXS. Schmitt and colleagues [13] used EEG to assess neural responses to self-generated speech sounds in adults with FXS, and unlike previous studies using nonsocial sounds, EEG responses in this study did not indicate cortical hyperactivation to sounds relative to controls, supporting a potential differential response to speech and nonspeech sounds in FXS.

Heightened responses to nonspeech sounds across multiple studies alongside no difference in response to speech sounds [13] may be associated with reduced cortical specialization for speech. Specifically, increased neural activity for nonspeech sounds may lead to a reduction in preferential activity for speech sound processing early in development. Importantly, An and colleagues [14] showed that hyperactivation to nonspeech sounds and reduced inhibition to repeated sounds are present in young children with FXS. Further, this pattern is associated with lower scores on both behavioral and parent-reported measures of language ability (i.e., the PLS-4 and Vineland [14]), . What remains to be seen is whether this pattern of responses to nonspeech sounds in FXS extends to speech sounds and if there is in fact cortical specialization for language in FXS.

In humans, cortical specialization for language is present at birth and develops extensively over the first few years of life and into childhood [15–17]. Two consistent features of cortical specialization in infants with typical development are differential activation to speech and nonspeech sounds [18, 19] and lateralization of response to speech in the left hemisphere [20, 21]. Neural differentiation of speech and nonspeech sounds emerges within the first year of life in typical development and predicts later language abilities as well as language impairments [22, 23]. This effect is present within days of birth [24], and is affected by prenatal auditory experiences [15].

Whereas there are no studies of neural differentiation of speech and nonspeech sounds in children with FXS, studies in both typical development and in other neurodevelopmental disorders suggest an important connection between the two. Specifically, early neural differentiation of speech sounds in infancy is associated with better language outcomes in typical development [22, 23]. However, lack of early cortical specialization for speech and language processing is present in autism [25] and is related to reduced social engagement [26]. The presence of neural differentiation of speech sounds in children with FXS is not known, but behaviorally, significant language delays emerge between 6 months and 2 years of age in the disorder [27]. Language delays in FXS are present across expressive and receptive language as well as pragmatic use of language in a social-communicative context [28]. While individuals with FXS do develop new language and communication abilities across development, delays in comparison to typical controls extend and widen into childhood, adolescence, and adulthood [29]. Areas of delay or difference include expressive, receptive, and pragmatic language as well as intelligibility of speech and articulation, as well as literacy [29]. Schmitt and colleagues showed a relation between neural responses to self-generated speech and both speech intelligibility and communicative abilities generally [13], with reduced pre-speech activity being associated with lower intelligibility and communicative abilities. However, the role of neural differentiation in childhood and the emergence of delays in FXS is unknown.

Regarding lateralization of auditory response, studies using functional MRI (fMRI) in FXS show hyperresponsiveness to nonspeech auditory stimuli in the left hemisphere auditory regions [30]. An MEG study using pure tones in FXS also showed an increased N1 amplitude following sounds, indicating a heightened neural response compared to controls very early in processing [31]. However, unlike the Hall et al. study, this heightened response was not lateralized. No fMRI or MEG studies report on neural activation to speech in FXS, nor are there currently any studies in children with FXS with spatial resolution sufficient for documenting cortical specialization for speech. An essential next step in understanding the neural basis for language delays in FXS is documenting cortical differentiation and lateralization of speech in the disorder. It is especially essential to investigate neural response to speech in FXS in childhood, to determine what (if any) facets of atypical neural response to speech relate to emerging delays.

Functional neural activity can be challenging to measure in children with FXS with techniques like fMRI and MEG because it can be difficult for individuals with sensory sensitivities, anxiety, and/or cognitive delays to tolerate the imaging environment while being still enough to obtain a reliable signal. Functional Near Infrared Spectroscopy (fNIRS) measures brain activity by relying on differential absorption of near infrared light in active and nonactive brain regions and can be measured with a wearable cap while a child is awake and somewhat active. Although not all individuals will tolerate wearing an fNIRS cap, its high signal to noise ratio and the subsequent ability to move freely during imaging leads to better tolerability for children, including those with neurodevelopmental disorders. fNIRS has been used to localize differential cortical responses to speech and nonspeech in typical development, and provides a robust, sensitive index of this differentiation in children and adults [32, 33]. fNIRs has also been used to track cortical specialization for language in infants with typical development [15, 23, 24], as well as in infants and toddlers at risk for neurodevelopmental disorders [34, 35]. fNIRS has been used to investigate face processing in individuals with FXS [36, 37], but has not been used to investigate sound perception, speech processing, or language in FXS to date.

Here, we use fNIRS in children with FXS to localize and characterize neural activation to speech and nonspeech sounds. Given the emergence of cortical specialization for speech and lateralization of activation in infancy, this first investigation of neural response to language in FXS focuses on activation to and differentiation of speech sounds. We hypothesize that children with FXS will vary from controls in both features; specifically, that they will show (a) reduced cortical differentiation of speech vs. nonspeech stimuli in the auditory cortex and (b) a less left-lateralized response to speech in comparison with a control group. This investigation is an important step in understanding the neural mechanisms for language and communication delays in FXS.

## Methods

This study was approved by the Institutional Review Board at Cincinnati Children’s Hospital Medical Center (CCHMC) and was performed in accordance with the protections set forth in the Declaration of Helsinki. Participant’s parents or legal guardians provided written informed consent for participation.

### Participants

52 children ages 2–10 years were recruited to participate in this study. Of those 52 children, 9 did not tolerate cap placement (17%, all FXS), two children with FXS completed the study but no event triggers were captured due to a recording error, and 3 children with FXS produced data that did not survive data processing cutoffs (e.g., *PruneChannels*, see below). The final sample therefore included 23 children with FXS (5 female) and 15 typically developing children (TDC, 4 female) ages 2–10 years, whose demographic data can be seen in Table 1. The groups were matched on sex, age, handedness, race, and ethnicity. Exclusion criteria for both groups included any documented history of hearing loss or parent concerns with hearing loss. Individuals in the control group had no history of psychiatric diagnosis or care, neurodevelopmental delay, early intervention, or special education placement. Recruitment occurred over a 2-year grant period, and the number of participants was determined by recruitment rates over that time, which overlapped with the COVID pandemic.

**Table 1:** Demographic data by group as well as assessment data for the FXS group

Variable (*n* FXS, *n* TDC)	FXS (23)	TDC (15)	difference
Sex M: F (23, 15)	18:5	11:4	Χ^2^ = 0.12, *p* = .73
Age (23, 15)	6.44 (1.90) [2.23–8.98]	7.07 (2.44) [2.18–10.71]	*t*(36)=-0.89, *p* = .38
Handedness (right: nonright) (23, 15)	16:7	12:3	Χ^2^ = 0.51, *p* = .48
White: Nonwhite (23, 15)	21:2	12:3	Χ^2^ = 1.01, *p* = .37
Hispanic: Nonhispanic (23, 14)	1:22	0:14	Χ^2^ = 2.2, *p* = .33
Vineland ABC (23, 13)	69.9 (16.8) [43–111]	107.8 (15.2) [78–133]	*t*(34) = 6.7, *p* < .0001
Verbal DQ	49.5 (19.6) [9.5–85.7]	N/A	N/A
ADOS Calibrated Severity Scores	5.6 (2.3) [1–10]	N/A	N/A

Note One person in the TDC group had missing data for ethnicity. Two individuals in the TDC group had missing data for the Vineland. FXS = Fragile X Syndrome, TDC = Typically Developing Control, DQ = Developmental Quotient, ADOS = Autism Diagnostic Observation Schedule, ABC = Adaptive Behavior Composite. The Vineland ABC is an age-normed standard score (SS), with a mean in the population of 100 and a standard deviation of 15

### Language measures

Parents of all participants but two (both TDC), completed the Vineland Adaptive Behavior Scales [VABS, 38], including the Expressive Language and Receptive Language subtests. The VABS is a parent-report measure of a child’s adaptive behaviors, or behaviors that they are able to execute consistently and independently in daily life. Here, the VABS v-scale scores, which have a mean of 15 and a standard deviation of 3 in the population, for the expressive and receptive language subtests were used to assess relation of fNIRS results to language in both the TDC and FXS groups. Most of the FXS participants (*n* = 21) and some of the TDC participants (*n* = 4) completed a cognitive evaluation, which included either the Mullen Scales of Early Learning [39] or the Stanford Binet 5th edition [40], dependent on age and ability. Language scores (either Mullen Receptive and Expressive Language scores or Stanford Binet Verbal scores) were converted into Developmental Quotients (DQs), because Standard Scores and T-scores make it challenging to study variation in individuals with developmental disabilities due to floor effects [41, 42]. We calculated DQs as (Age Equivalent/Chronological Age)*100. DQs were used to evaluate the relation between fNIRS results and language in the FXS group but not the TDC group due to low completion in the TDC group.

### Other measures

Handedness was evaluated using the Edinburgh Handedness Inventory for children ages 6 and up [43] and via parent report in younger children. Presence of autism symptoms was evaluated in individuals with Fragile X Syndrome using the Autism Diagnostic Observation Schedule, 2nd edition [ADOS-2, 44]. The ADOS is a play and interaction-based measure that was completed and scored by a clinical psychologist with expertise in autism symptoms in the context of Fragile X Syndrome. The calibrated severity score ranges from 1 to 10 with higher scores indicating higher severity of autism symptoms during the ADOS evaluation [45].

### fNIRS system

**Fig. 1:**
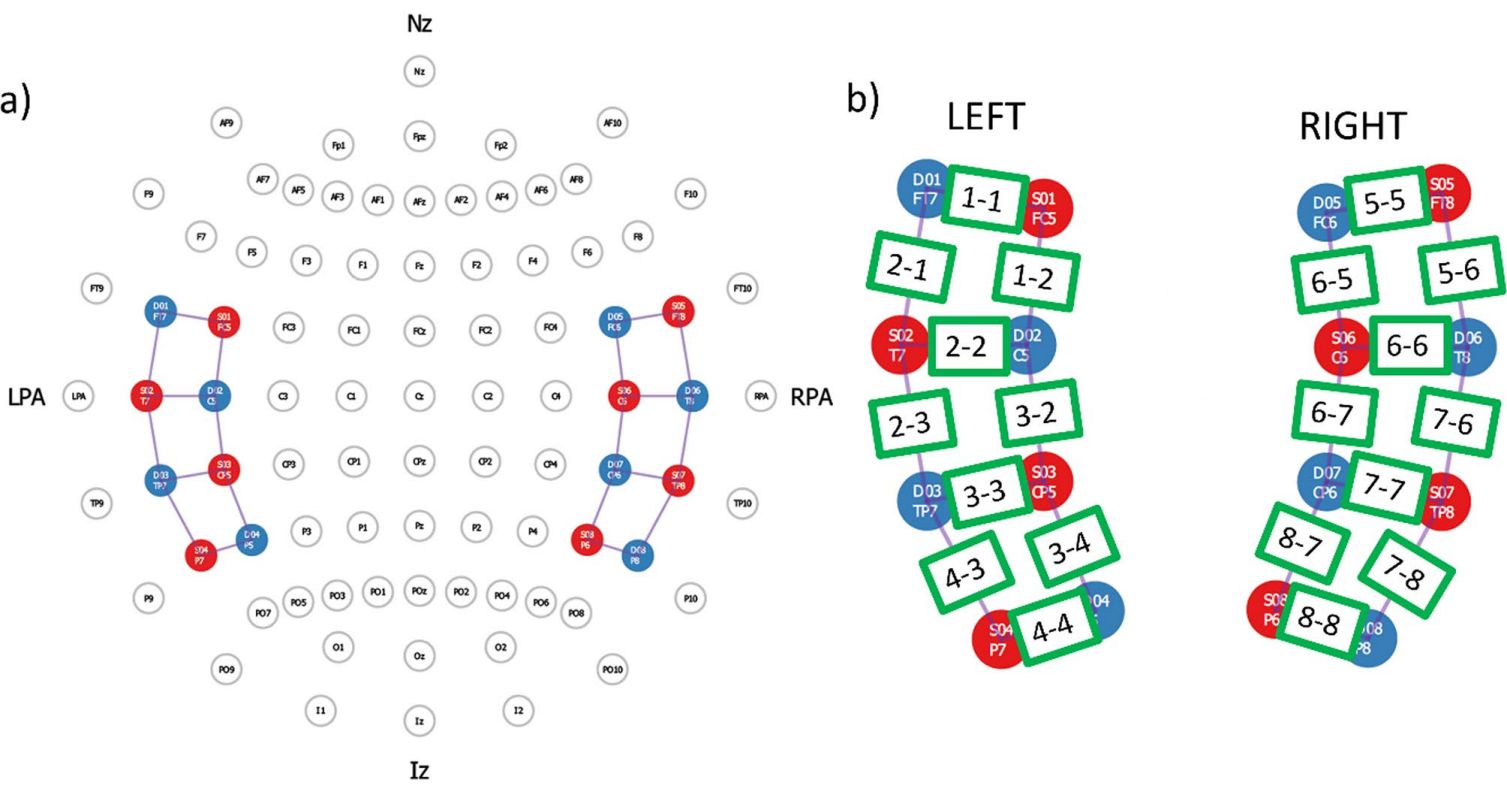
FNIRS optode layout and channel locations. *Note* Image created using NIRSSite2020.7. **a**) shows optode layout in the context of International 10–20 locations. **b**) shows optode layout closeup, with red circles representing sources, blue circles representing detectors, and green rectangles representing channels. Nomenclature for channels is Source number- Detector number

All fNIRS data were collected using a custom NIRx NIRScoutX 64 × 32(NIRx Medical Technologies, Brooklyn, NY) machine with lasers at wavelengths of 685 and 830 nm and a 7.8125 Hz sampling frequency. The probe layout was an auditory 2 × 2 × 4 montage designed to cover temporal lobe areas bilaterally including the primary auditory cortex (see Fig. 1 for visualization of probe layout and cap design). This exact layout has been used in other fNIRs studies focused on the auditory cortex [46, 47]. Probes were arranged in a stretchy cap with International 10–20 markings, with cap size based on same-day head circumference measurements for each child. Distances between light emitters and detectors varied between 2 and 3 cm, dependent on cap size and head shape. A total of 20 channels were analyzed (10 on each side). Data were collected using NIRStar 15.2 acquisition software with auditory stimuli programmed and presented using via NIRStim software within the NIRStar system.

### Paradigm

**Fig. 2:**
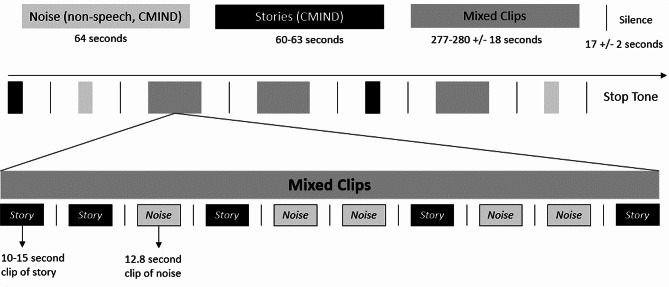
Passive auditory listening paradigm. Note Only data from “Mixed Clips” were analyzed here

The experimental design was a passive listening task lasting approximately 20 min (variable due to jitter, actual time was 1,208 s plus or minus up to 68 s of jitter). Participants were seated in a chair independently or on a parent’s lap in front of a desk with a computer monitor and two speakers. Standardized non-social video clips (e.g., factory scenes, dominos, non-social animations, etc.) were presented on the video monitor to reduce boredom without interfering with the auditory task. The auditory task consisted of a modified version of the Stories paradigm created by Cincinnati MR Imaging of Neurodevelopment (C-MIND), which is a passive listening paradigm presented to participants without instructions [48, 49]. The original paradigm consists of 5 recorded stories in a female voice each lasting about 1 min in length (60–63 s) and a nonspeech condition lasting 64 s. The nonspeech condition consists of broadband noise, presented in sweep format. Center frequencies are set at 2000 to 4000 Hz in order to mimic representation of frequency variation in speech, and sweep durations similarly varied between 0.5 and 2.0 Hz. Participants in the present study heard both examples of these original 1-minute clips (not analyzed here) as well as modified (i.e., shortened) clips. The shortened clips were modified to match block designs typically used in fNIRS studies, while the longer stories were recorded (but not analyzed in the present paper) for comparison to fMRI literature. Specifically, for the nonspeech condition the 64 s stimulus was divided into shorter, 12.4 s segments. The stories were divided into 5 segments, ranging from 10 to 14 s, such that segmentation did not alter words or clauses. The overall task was then presented as a block design in which each block was presented in a random order (see Fig. 2). There were 2 blocks with a full story recording (stimulus length = ~ 60 s), 2 blocks with full nonspeech stimulus presented (stimulus length = 60 s), and 3 mixed blocks with stimulus length of speech and nonspeech stimuli varying between 10 and 14 s (which are analyzed in the present study). In the mixed blocks, both the stories and nonspeech segments are randomly interspersed (with stories presented in chronological order), with a total of 15 story segments and 15 nonspeech segments. Between each block and each segment there was 17 +/- 2 s of jittered silence. In each of the 3 mixed blocks the nonspeech clips were pseudorandomly ordered, while shortened story clips were played in consecutive order (i.e., in story order). Five orders were presented to participants such that each of the 5 stories was represented in the full story and story segment conditions between subjects. The present study uses data acquired for the mixed clips (i.e., 30 total blocks, 10–14 s each). All sounds were presented at the same mean sound intensity across participants (determined to be comfortable during study piloting), and sound intensity was also matched between the speech and nonspeech conditions.

### fNIRS data processing

Raw data was converted to SNIRF format in Matlab using the NIRS2Snirf function. Data were then processed using the Homer3 toolbox (https://github.com/BUNPC/Homer3). First, data recorded prior to the first marker and after the last marker were removed manually. Then, after converting data from Intensity to Optical Density, PruneChannels was applied to remove noisy channels from individual data, with settings as follows: drange=[0.05 5], SNRthresh = 5, SDrange=[0 45]. This function detects and then removes an entire channel from an individual participant’s data across all trials. Three individuals, all with FXS, did not have any data remaining after PruneChannels, so their data is not represented in this dataset. Thus, all 23 individuals with FXS and 15 individuals in the TDC group had at least one channel of usable data remaining (mean = 16 channels per participant, minimum = 3, maximum = 20). Number of usable channels did not vary by group (t = 0.003, *p* = .997). After using hmrR_MotionArtifactByChannel (standard settings) to detect motion artifact, we used hmrR_MotionCorrectWavelet (standard settings) to correct detected artifact in the Optical Density data. Use of this combination of functions means that data with detected motion artifact is not removed but is instead corrected. Finally, we applied a lowpass filter at 0.5 hz, to reduce contamination with heartrate signals, converted data to Concentration of Hb and HbO molecules in µmol, and exported all Hb and HbO values as well as mean values by condition (speech, nonspeech) across blocks, defined as beginning 2s before the stimulus and ending 20s after stimulus onset. Before being analyzed, mean Hb and HbO values that were greater than 3 standard deviations from the mean for the study were excluded as outliers (total data points removed = 18 out of a possible 2392).

### Data analysis

We used a linear mixed effect model (lme4 in R) to predict mean values for Oxy and Deoxygenated hemoglobin concentration based on group and condition (speech, nonspeech), while controlling for age and sex. We chose to control for age and sex because, although both are matched in this sample, both are known to relate to neural activation to speech and nonspeech sounds. Analyses were also completed without accounting for age and sex, and can be seen in Supplementary Information. Specifically, group membership (FXS vs. TDC) and condition (speech vs. nonspeech) predicted changes in Oxy and Deoxygenated hemoglobin concentration from baseline while subject ID was entered as a random factor to account for within-subject variability. The linear mixed effects model, with subject ID as the random factor, allows for analysis of repeated measurements (e.g., multiple channels and conditions) while controlling for variability within subject ID. It also allows for inclusion of data when channels or blocks are limited by data loss (as is common in pediatric neuroimaging) by including all clean data. While each model can therefore include multiple data points from one participant, significance tests for that model are from the pooled data across all participants. In order to characterize changes in blood flow related to condition, we first identified those channels within each group that showed significant relative increases in HbO and decreases in Hb during those conditions compared to baseline. In order to measure neural discrimination, we examined effects of condition (speech vs. nonspeech) on neural activity compared to baseline at each of 20 channels within each group (e.g., the effect of condition on the relative changes in Hb and HbO concentration from baseline). The effect of condition was also determined within the multilevel framework to account for individual variability in hemoglobin levels, preventing individuals with higher oxygenation levels generally (which can be related to skull thickness, skin tone) to drive analyses. To investigate lateralization patterns, we investigated the effect of side (left vs. right) on neural activity within the speech condition for each group. Finally, in order to investigate relation between any effects and clinical variables, we investigated interactions between the clinical variable (i.e. Vineland Receptive and Expressive scores, Verbal Developmental Quotient) and the specific effect within the linear mixed effects model. To correct for multiple comparisons, we used a 5% false discovery rate (FDR) cutoff across all analyses (total number of tests = 167).

## Results

### Activation for speech and nonspeech in controls and in FXS

**Fig. 3:**
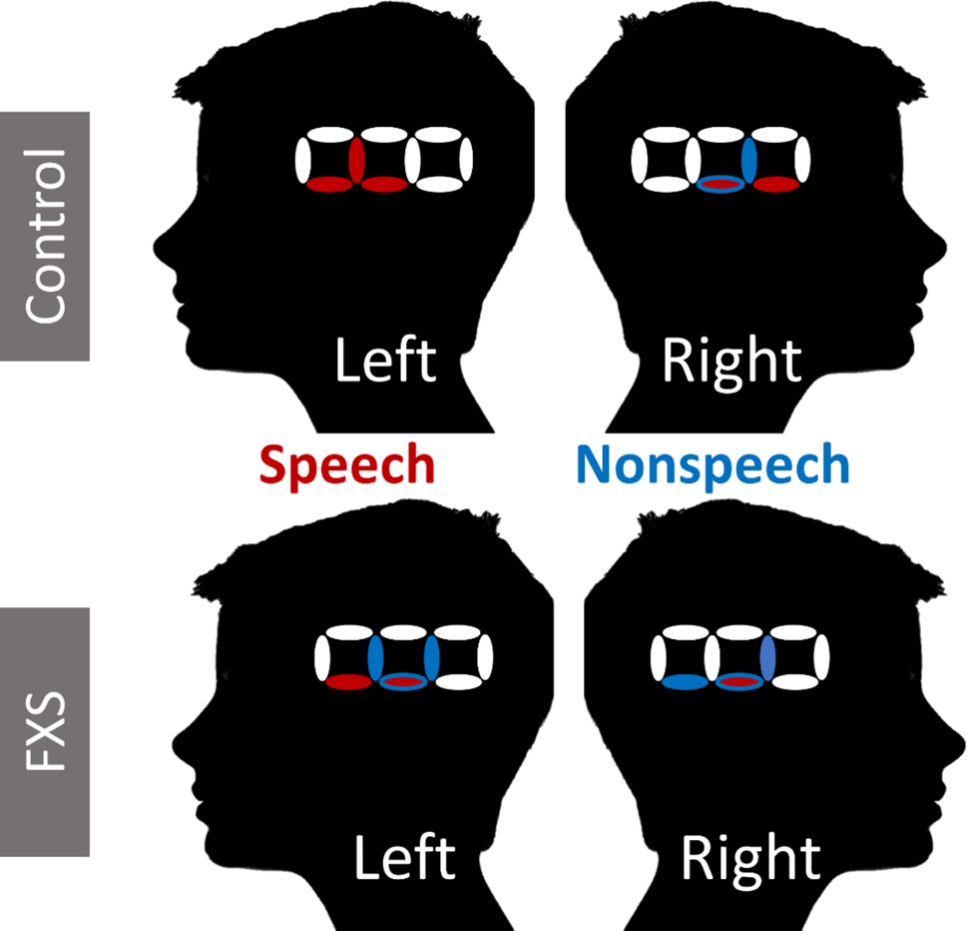
Channels with significant activation for speech and nonspeech in the FXS and Control Groups. Note Neural activation for speech stimuli are indicated in red, while activation for nonspeech stimuli are indicated in blue

Controls showed significant activation to speech in two left hemisphere channels (channel 2_1 t(13)=-3.21, *p* = .004; channel 2_3 t(11)=-2.64, *p* = .017, see Fig. 3) with marginally significant activation in one left hemisphere channel (channel2_2 t(11)=-2.01, *p* = .052). Controls showed activation to speech in two right hemisphere channels as well (channel 5_6, t(14)=-2.1, *p* = .046, channel 7_6 t(14)=-3.78, *p* = .002). Controls showed activation to nonspeech in two right hemisphere channels (channel 6_6 t(13)=-2.23, *p* = .046; channel 7_6 t(14)=-3.79, *p* = .0002). When looking across all 20 channels, the TDC group did not show an effect of side (left vs. right) on activation to the speech stimuli (t(15) = 0.70,*p* = .49) or nonspeech stimuli (t(15)=-0.67, *p* = .50).

Individuals with FXS, however, showed activation to speech in two left hemisphere channels (channel 2_1 t(20)=-3.06, *p* = .0065; channel 2_3 t(18)=-3.72, *p* = .0017) and in one right hemisphere channel (channel 7_6 t(19)=-3.02, *p* = .007, see Fig. 3). Individuals with FXS showed activation to nonspeech sounds across three left hemisphere channels (channel 2_2 t(18)=-2.97, *p* = .006; channel 2_3 t(18)=-3.60, *p* = .0022; channel 3_3 t(14)=-2.36, *p* = .035). In the right hemisphere, individuals with FXS showed activation for nonspeech sounds across three channels (channel 6_6 t(19)=-2.12, *p* = .04; channel 7_6 t(19)=-2.18, *p* = .036; channel 7_8 t(18)=-2.03, *p* = .05). Individuals with FXS did show greater activation in the left compared to right cortex for speech when looking across all 20 channels (t(22) = 2.43, *p* = .015) but did not show an effect of side on activation for nonspeech stimuli (t(23)=-0.16, *p* = .88).

After correction for multiple comparisons, the only significant effects remaining included the TDC response to nonspeech in channel 7_6 and the FXS response to speech in channel 2_3.

### Discrimination of speech and nonspeech sounds

**Fig. 4:**
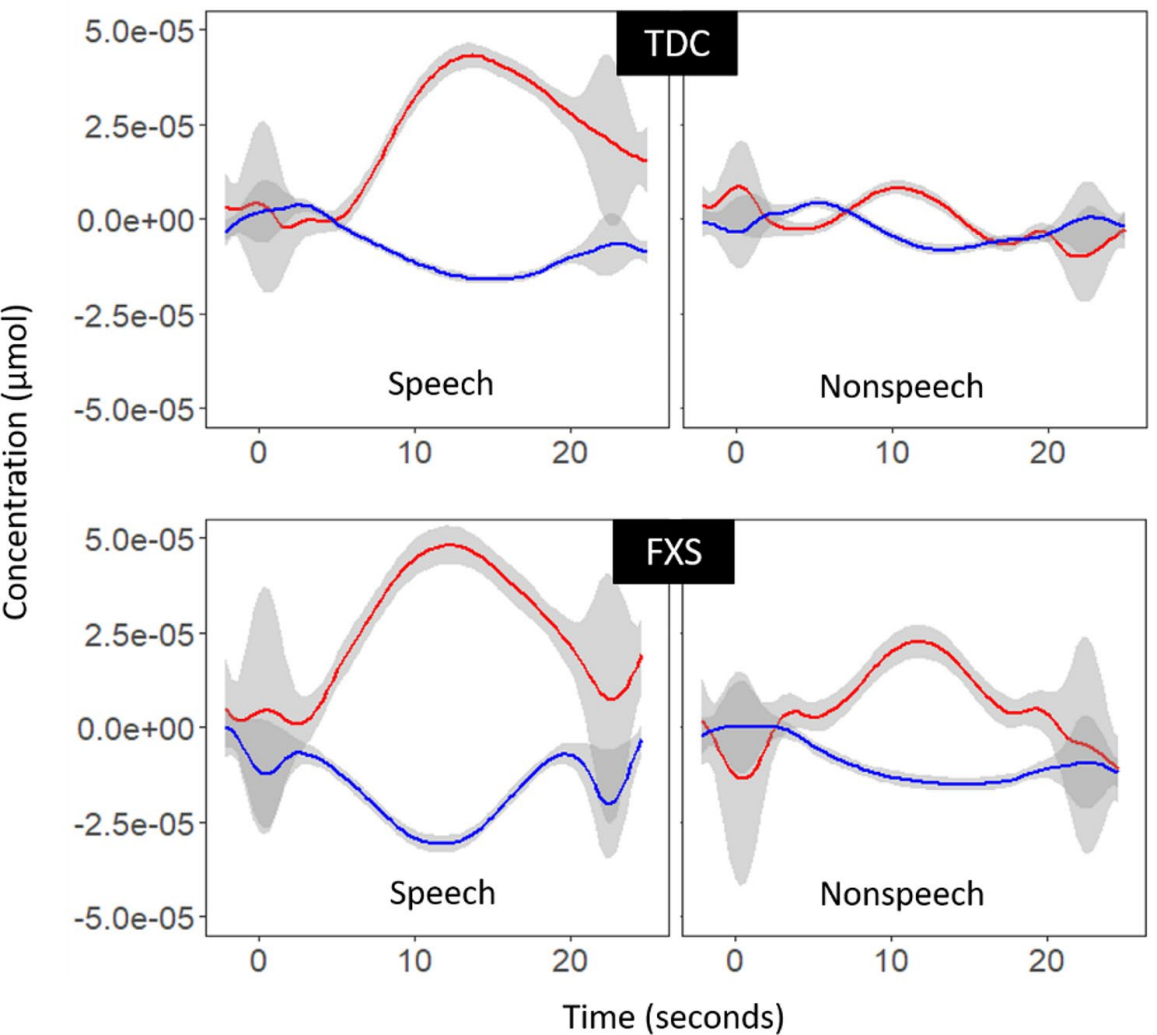
Differential response to speech and nonspeech between groups at Channel 2_1 in left auditory cortex. Note Red lines represent change in concentration of Oxygenated hemoglobin over time in comparison to the baseline, while blue lines represent concentration of Deoxygenated hemoglobin. Grey areas represent 95% confidence interval of concentration at each time point

We located channels where activation for the speech condition and for the nonspeech condition varied significantly by determining relative differences in changes in Hb and HbO from baseline by condition (speech vs. nonspeech). Controls showed neural discrimination in the lateral most anterior channel in the left hemisphere (channel 2_1, between 10 and 20 coordinates T7 and FC7, t(13)=-2.71,*p* = .01, Cohen’s d = 1.5). Examination of waveforms shows greater activation for speech versus nonspeech in TDC (Fig. 4). Individuals in the TDC group did not show differential discrimination of speech and nonspeech stimuli by hemisphere (left, right) across all 20 channels (t(15) = 0.95, *p* = .34) but did show greater differentiation of speech and nonspeech in the left versus right hemisphere at the channel level (for channels 2_2 versus 6_6, t(13) = 2.30, *p* = .024, Cohen’s d = 1.28).

Individuals with FXS did not show significant discrimination of speech vs. nonspeech sounds in any channel (e.g., channel 2_1, t(20=-1.35, *p* = .18, Cohen’s d = 0.6)). They did not show differential discrimination by hemisphere across all channels (t(23) = 1.735, *p* = .08, Cohen’s d = 0.72), and did not show hemispheric effects within channel pairs across the left and right hemispheres (all p’s > 0.05).

Neither finding in the TDC group (discrimination of speech and nonspeech at channel 2_1 nor hemispheric differences in differentiation at channels 2_2 and 6_6) remained significant after correction for multiple comparisons.

### Post hoc

Analyses at individual channels showed robust activity for the nonspeech condition in FXS compared to controls, as seen in Fig. 3. The FXS group showed greater activation for the nonspeech condition than TDC across the left and right auditory cortex combined (t(38) = 2.47,*p* = .014), which was also significant within the right hemisphere (t(38) = 2.02, *p* = .04), but not the left hemisphere (t(38) = 1.865, *p* = .06). There was no group difference in nonspeech processing within any specific channels (all p’s > 0.05). Differential processing of nonspeech in FXS vs. TDC across the auditory cortex did not remain significant after correction for multiple comparisons.

### Relation to language ability

We investigated the relation between significant findings noted above and language measures separately for the FXS and TDC group (Table 2). Within the FXS group, neural discrimination of speech and nonspeech at channel 2_1 (where TDC group showed neural discrimination) did not predict scores of any language measures (for Verbal DQ, t(17)=-0.52, *p* = .608; Vineland Receptive Language, t(20)=-1.27, *p* = .21; Vineland Expressive Language, t(20)=-0.49, *p* = .62). Degree of neural lateralization for speech across all channels was not predicted by Verbal DQ (t(19) = 1.472, *p* = .141). However, Vineland Receptive scores were associated with lateralization for speech, while Vineland Expressive scores were nominally associated (for VABS receptive, t(22) = 2.28, *p* = .02; for VABS expressive t(22) = 1.93, *p* = .05). Specifically, higher parent-report of receptive and expressive language ability was associated with greater leftward lateralization for speech stimuli in FXS. Increased response to nonspeech stimuli across all 20 channels was not significantly correlated with Verbal DQ (t(20) = 1.89, *p* = .059),Vineland Receptive or Expressive language scores (t(23) = 0.58, *p* = .56; t(23)=-1.34, *p* = .18). The relation between Vineland language scores and leftward lateralization of speech stimuli did not remain significant after correction for multiple comparisons (for RL, Cohen’s d = 0.97; for EL, d = 0.82).

In the TDC group, we investigated the relation between the above findings and scores on the Vineland receptive and expressive subscales, but did not investigate relation to Developmental Quotient due to missing data (TDC n with DQ data = 5). For the TDC group, neural discrimination of speech and nonspeech stimuli at channel 2_1 was not associated with Vineland receptive language (t(11)=-0.69, *p* = .49), but was associated with Vineland expressive language (t(11)=-2.06, *p* = .05, Cohen’s d = 0.67). Lateralization for the speech condition was not associated with Vineland receptive language (t(13) = 1.09, *p* = .28), but was associated with Vineland expressive language (t(13) = 2.02, *p* = .04). Specifically, higher parent report of expressive language use was associated with increased discrimination of speech and nonspeech and with increased leftward lateralization for speech. Response to the nonspeech stimulus across the brain was not associated with either expressive or receptive language on the Vineland for the TDC group (receptive t(13) = 0.54, *p* = .59; expressive t(13) = 0.57, *p* = .57). The relation between Vineland expressive language scores and both neural discrimination and leftward lateralization of speech stimuli did not remain significant after correction for multiple comparisons (for discrimination, Cohen’s d = 0.97; for lateralization, d = 0.82).

**Table 2:** Summary of associations between language and neural measures in FXS and TDC groups

	Discrimination of speech and nonspeech stimuli at channel 2_1	Lateralization for speech stimuli	Response to Nonspeech stimuli
Vineland Expressive subscale	FXS *n.s.* **TDC*p*** **= 0.05**	**FXS*p*** **= 0.05** **TDC*p*** **< 0.05**	FXS *n.s.* TDC *n.s.*
Vineland Receptive subscale	FXS *n.s.*TDC *n.s.*	**FXS*p*** **< 0.05** TDC *n.s.*	FXS *n.s.* TDC *n.s.*
Verbal DQ	FXS *n.s.* TDC N/A	FXS *n.s.* TDC N/A	FXS *n.s.* TDC N/A

## Discussion

In this study, we demonstrate for the first time localized cortical activation to speech in children with FXS. We also investigate for the first time differences in neural differentiation between speech and nonspeech stimuli in children with FXS. Both individuals with FXS and controls showed brain activation to both speech and nonspeech stimuli within the auditory cortex as well as left-lateralizated responses, with lateralized response to speech in FXS and lateralized discrimination of speech and nonspeech in TDC. The main differences between the two groups were (1) lack of neural differentiation between speech and nonspeech in FXS and (2) our post hoc finding of greater response to nonspeech in FXS. This is an important step toward understanding how FXS affects neural representation of language processing and indicates an important role for understanding nonspeech stimuli in early development in FXS.

In the present study, we show neural discrimination of speech and nonspeech sounds (with similar low frequency modulations) in a small group of control children across a very wide age range (2–10 years). This effect was observed at one channel in the left auditory cortex. In FXS, lack of differentiation of speech and nonspeech stimuli, especially if present during the first years of life, would indicate early emerging differences in language processing. If lack of differentiation of speech and nonspeech sounds in the early years leads to reduced attention afforded to speech in FXS, this could also lead to a cascade of further speech and language impairments. Another possible mechanism is that the brain in FXS may be affording increased attention to nonspeech auditory stimuli. This is supported by the extant EEG literature in FXS as outlined earlier and could precede differences in neural discrimination during development. An important next step for this literature will be assessment of neural discrimination of speech and nonspeech sounds in FXS in the first years of life.

Regarding neural lateralization to speech and nonspeech sounds in the present study, some research suggests that the left hemisphere is the locus of processing for sound changes that are in the higher frequency ranges, while the right hemisphere specializes in lower frequency changes in sound presentations [50]. Here, our speech signal contained complex vocal information, with sounds varying in the phonetic, phonologic, morphologic, and syntactical levels. Importantly, the nonspeech condition was presented in sweep format, with sweeps presented at 0.5-2 hz, which mimics those variations in speech at the morphologic/syntactical levels. This control allows us to more precisely locate regions associated with understanding language rather than those auditory regions that detect slower temporal changes. However, this also means that our nonspeech condition mimics certain aspects of speech, and therefore would be expected to activate regions associated particularly with processing lower frequency variations in speech. Therefore, we might expect activations of regions specializing in sound changes within the slower frequencies for the nonspeech condition. It is possible that responses to the nonspeech condition in the right hemisphere in particular in both the TDC and FXS groups in the present study are related to the ways that this condition mimics slower variations present in speech. Given these possible ‘speech-like” features, future studies focused on nonspeech sound perception in FXS, including hyperactivation to nonspeech sounds, will need to measure neural response to sounds without speech-like features (e.g., environmental sounds).

While speech perception was represented across both left and right auditory cortices in both FXS and TDC in the present study, activation for speech was stronger in the left hemisphere for the FXS group, while discrimination of speech and nonspeech was stronger in the left hemisphere for the TDC group. This provides some evidence that lateralized responses to speech stimuli are preserved in FXS. As predicted by the lower frequency variations in the nonspeech stimuli, the control group showed specialized neural activity to these stimuli within the right hemisphere temporal regions. What is perhaps most interesting about lateralization in the FXS brain in this case, is the strong response to the nonspeech, slowly varying sounds within the left hemisphere temporal regions. Future studies would need to clarify the role of frequency of sound variations to determine if this pattern represents a link between atypical neural organization and language delays in FXS.

We had predicted that the FXS group would show reduced lateralization based on the literature showing differential lateralization of speech in other neurodevelopmental disorders, including autism [51, 52]. However, our results showed lateralization for speech perception (although not discrimination) at the level of the blood oxygen level dependent response. It is possible that differences in lateralization may be detectable within smaller timescales, such as with event related potentials [31]. While we do not compare lateralization between groups here, other studies of FXS have in fact shown increased leftward lateralization for sound processing in adolescents and adults with FXS [8, 30]. Given that lateralization patterns change across childhood in typical development, it will be important for future studies investigating lateralization in FXS to use a longitudinal framework.

In this study, individuals with FXS showed greater neural activation to the nonspeech condition than controls across left and right auditory cortex. This effect may be related to temporal features present in the nonspeech condition as described above, and future studies investigating neural activation for speech filtered with varying levels of temporal information in FXS will be an important next step. It is also possible that the response to the nonspeech here is an activation to “noise”, and may in fact provide a neural analog for the well-documented effects of hyperacusis in Fragile X Syndrome [3]. An important next step for investigating this possibility will be investigating how this effect relates to sensory processing phenotypes within FXS as well as how it relates to neural activation related to auditory discomfort. Additionally, future research should include infants and toddlers with FXS in order to understand how neural response to nonspeech sounds affects early language development in FXS.

While this study is the first report of localized neural activation to speech in FXS, there are several limitations that should be considered in interpreting the above findings. First, this study is cross sectional and covers a broad age range within the context of “childhood”, and it is not possible to determine developmental effects within this sample [53]. Cross sectional age was controlled for in all analyses, but this does not eliminate problems that could emerge with an interaction between age and any of the target variables. This is especially problematic given evidence that auditory processing differences may vary by age in the mouse model of FXS [54]. An essential next step for this literature will include longitudinal studies of a smaller age range of children with FXS. Second, when using fNIRS it can be difficult to disentangle cortical effects from those driven by other physiological sources (e.g., increased respiration rate, increased arousal). In this study, data were filtered to remove heart rate, but other sources can contaminate the signal, making it difficult to determine the degree to which effects can be driven by arousal. An important next step for this work will be measurement and targeted removal of physiological noise. Additionally, whereas localization of cortical activity via fNIRS has been validated through concurrent fMRI studies [55, 56], exact localization is not possible to determine for each participant without completing a structural brain scan that is individually matched to 3D fNIRS channel location information. Although activation patterns, especially across the well-studied 10–20 locations [57], can be useful in localizing brain activity, identification of specific cortical regions with spatial resolution in the millimeter range will require future studies using both fNIRS (including 3D localization of precise optode locations) and structural MRI. In addition, given evidence that auditory processing differences may exist beyond the auditory cortex in the mouse model of FXS [54], future fNIRS studies of auditory processing in FXS should extend coverage to the frontal lobe. Finally, while this study is the first to investigate neural response to speech in children with FXS, it may not have been powered to detect neural discrimination in the FXS population. This is especially the case given the number of statistical tests run in this initial investigation, resulting in lack of significant findings after FDR correction. Based on the present study, neural discrimination in left auditory cortex in FXS would be expected to have a medium effect size (Cohen’s d = 0.6). With power set at 0.8 and probability at 0.05, a sample size of 45 children with FXS is recommended for future studies of neural discrimination of speech and nonspeech sounds. An increased sample size across both groups might also make activation patterns more robust across the auditory cortex, and would make it possible to determine a more clear relation between neural activation and neural discrimination during speech sound processing in FXS. It will also be important for future studies of these neural patterns to establish a clear link with language development within typical development, including more extensive phenotyping in this group, in order to ground relations between neural activity and language measures in FXS. Finally, determining specificity of these findings to FXS will require comparison to groups of children matched on intellectual ability to the FXS group.

This first study of localization of speech perception in FXS is an important next step in understanding how early language delays emerge in FXS. We showed intact lateralization of speech sound activation in FXS within the left auditory cortex. However, individuals with FXS showed a stronger response to the nonspeech condition than controls and did not discriminate between speech and nonspeech stimuli. This brings the role of sound processing in FXS and its effects on speech and language development into focus and provides an avenue for future exploration.

## Electronic supplementary material

Below is the link to the electronic supplementary material.

Supplementary Material 1

## Data Availability

The dataset supporting the conclusions of this article is available in the zenodo repository and can be accessed through the following link: https://zenodo.org/records/10384556?token=eyJhbGciOiJIUzUxMiJ9.eyJpZCI6IjhjMjZiZDQ0LTlhNDctNGU5NC05Zjk5LTlkMzQ4MzU3NTRhMCIsImRhdGEiOnt9LCJyYW5kb20iOiJkNzMyYjFiYThkYjM1ODkxMmMwNmNmZTg5MTFmNGY0OCJ9.U2eeuPUUDLdS29jQrtxYzTHPY42kXGUCDvewMP8CcxhxL3D5uyzR3_i6e1NkMN-OeWC9YbEv-objYBzq-Qk6HQ.

## References

[1] Abbeduto L, Brady N, Kover ST. Language development and fragile X syndrome: profiles, syndrome-specificity, and within-syndrome differences. Ment Retard Dev Disabil Res Rev. 2007;13(1):36–46. 17326110 10.1002/mrdd.20142 PMC7416600

[2] Wen TH, Lovelace JW, Ethell IM, Binder DK, Razak KA. Developmental changes in EEG phenotypes in a mouse model of Fragile X Syndrome. Neuroscience. 2019;398:126–43. 30528856 10.1016/j.neuroscience.2018.11.047 PMC6331246

[3] McCullagh EA, Rotschafer SE, Auerbach BD, Klug A, Kaczmarek LK, Cramer KS, Kulesza RJ Jr., Razak KA, Lovelace JW, Lu Y, Koch U, Wang Y. Mechanisms underlying auditory processing deficits in Fragile X syndrome. Faseb j. 2020;34(3):3501–18. 32039504 10.1096/fj.201902435R PMC7347277

[4] Pfeiffer BE, Huber KM. The state of synapses in Fragile X Syndrome. Neuroscientist. 2009;15(5):549–67. 19325170 10.1177/1073858409333075 PMC2762019

[5] Comery TA, Harris JB, Willems PJ, Oostra BA, Irwin SA, Weiler IJ, Greenough WT. Abnormal dendritic spines in fragile X knockout mice: maturation and pruning deficits. Proc Natl Acad Sci U S A. 1997;94(10):5401–4. 9144249 10.1073/pnas.94.10.5401 PMC24690

[6] Rotschafer SE, Marshak S, Cramer KS. Deletion of Fmr1 alters function and synaptic inputs in the Auditory Brainstem. PLoS ONE. 2015;10(2):e0117266. 25679778 10.1371/journal.pone.0117266 PMC4332492

[7] Rotschafer S, Razak K. Altered auditory processing in a mouse model of fragile X syndrome. Brain Res. 2013;1506:12–24. 23458504 10.1016/j.brainres.2013.02.038

[8] Van der Molen MJW, Van der Molen MW, Ridderinkhof KR, Hamel BCJ, Curfs LMG, Ramakers GJA. Auditory change detection in fragile X syndrome males: a brain potential study. Clin Neurophysiol. 2012;123(7):1309–18. 22192499 10.1016/j.clinph.2011.11.039

[9] Castrén M, Pääkkönen A, Tarkka IM, Ryynänen M, Partanen J. Augmentation of auditory N1 in children with fragile X syndrome. Brain Topogr. 2003;15(3):165–71. 12705812 10.1023/a:1022606200636

[10] Ethridge L, Thaliath A, Kraff J, Nijhawan K, Berry-Kravis E. Development of neural response to Novel sounds in Fragile X Syndrome: potential biomarkers. Am J Intellect Dev Disabil. 2020;125(6):449–64. 33211818 10.1352/1944-7558-125.6.449 PMC8631234

[11] Ethridge LE, De Stefano LA, Schmitt LM, Woodruff NE, Brown KL, Tran M, Wang J, Pedapati EV, Erickson CA, Sweeney JA. Auditory EEG Biomarkers in Fragile X Syndrome: Clinical Relevance. Frontiers in Integrative Neuroscience, 2019. 13(60). 10.3389/fnint.2019.00060 PMC6794497 31649514

[12] Ethridge LE, White SP, Mosconi MW, Wang J, Byerly MJ, Sweeney JA. Reduced habituation of auditory evoked potentials indicate cortical hyper-excitability in Fragile X Syndrome. Translational Psychiatry. 2016;6:e787. 27093069 10.1038/tp.2016.48 PMC4872406

[13] Schmitt LM, Wang J, Pedapati EV, Thurman AJ, Abbeduto L, Erickson CA, Sweeney JA. A neurophysiological model of speech production deficits in fragile X syndrome. Brain Commun, 2020. 2(1). 10.1093/braincomms/fcz042 PMC7425415 32924010

[14] An WW, Nelson CA, Wilkinson CL. Neural response to repeated auditory stimuli and its association with early language ability in male children with Fragile X syndrome. Front Integr Neurosci. 2022;16:987184. 36452884 10.3389/fnint.2022.987184 PMC9702328

[15] May L, Byers-Heinlein K, Gervain J, Werker J. Language and the newborn brain: does prenatal Language experience shape the neonate neural response to Speech? Front Psychol, 2011. 2. 10.3389/fpsyg.2011.00222 PMC3177294 21960980

[16] Perani D, Saccuman MC, Scifo P, Anwander A, Spada D, Baldoli C, Poloniato A, Lohmann G, Friederici AD. Neural language networks at birth. Proceedings of the National Academy of Sciences of the United States of America, 2011. 108(38): pp. 16056–16061. 10.1073/pnas.1102991108 PMC3179044 21896765

[17] Scheinost D, Chang J, Lacadie C, Brennan-Wydra E, Constable RT, Chawarska K, Ment LR. Functional connectivity for the language network in the developing brain: 30 weeks of gestation to 30 months of age. Cereb Cortex. 2022;32(15):3289–301. 34875024 10.1093/cercor/bhab415 PMC9340393

[18] Blasi A, Mercure E, Lloyd-Fox S, Thomson A, Brammer M, Sauter D, Deeley Q, Barker GJ, Renvall V, Deoni S, Gasston D, Steven CR, Williams MH, Johnson A, Simmons, Declan GM, Murphy. Early specialization for Voice and emotion Processing in the infant brain. Curr Biol. 2011;21(14):1220–4. 21723130 10.1016/j.cub.2011.06.009

[19] Lloyd-Fox S, Blasi A, Mercure E, Elwell CE, Johnson MH. The emergence of cerebral specialization for the human voice over the first months of life. Soc Neurosci. 2012;7(3):317–30. 21950945 10.1080/17470919.2011.614696

[20] Telkemeyer S, Rossi S, Nierhaus T, Steinbrink J, Obrig H, Wartenburger I. Acoustic Processing of Temporally Modulated Sounds in Infants: Evidence from a Combined Near-Infrared Spectroscopy and EEG Study. Frontiers in Psychology, 2011. 2(62). 10.3389/fpsyg.2011.00062 PMC3110620 21716574

[21] Shultz S, Vouloumanos A, Bennett RH, Pelphrey K. Neural specialization for speech in the first months of life. Dev Sci. 2014;17(5):766–74. 24576182 10.1111/desc.12151 PMC4232861

[22] Zhao TC, Boorom O, Kuhl PK, Gordon R. Infants’ neural speech discrimination predicts individual differences in grammar ability at 6 years of age and their risk of developing speech-language disorders. Dev Cogn Neurosci. 2021;48:100949–100949. 33823366 10.1016/j.dcn.2021.100949 PMC8047161

[23] McDonald NM, Perdue KL, Eilbott J, Loyal J, Shic F, Pelphrey KA. Infant brain responses to social sounds: a longitudinal functional near-infrared spectroscopy study. Dev Cogn Neurosci. 2019;36:100638. 30889544 10.1016/j.dcn.2019.100638 PMC7033285

[24] Cabrera L, Gervain J. Speech perception at birth: the brain encodes fast and slow temporal information. Sci Adv. 2020;6(30):eaba7830. 32832669 10.1126/sciadv.aba7830 PMC7439442

[25] Eyler LT, Pierce K, Courchesne E. A failure of left temporal cortex to specialize for language is an early emerging and fundamental property of autism. Brain. 2012;135(3):949–60. 22350062 10.1093/brain/awr364 PMC3286331

[26] Blasi A, Lloyd-Fox S, Sethna V, Brammer MJ, Mercure E, Murray L, Williams SCR, Simmons A, Murphy DGM, Johnson MH. Atypical processing of voice sounds in infants at risk for autism spectrum disorder. Cortex. 2015;71:122–33. 26200892 10.1016/j.cortex.2015.06.015 PMC4582069

[27] Emergence of Developmental Delay in Infants and Toddlers With an FMR1Mutation. Pediatrics, 2021. 147(5): p. e2020011528. 10.1542/peds.2020-011528 PMC8086007 33911031

[28] Mattie LJ, Hamrick LR. Early communication development in infants and toddlers with Fragile X syndrome. Autism Dev Lang Impairments. 2022;7:23969415221099403. 10.1177/23969415221099403 PMC9685137 36438157

[29] Hoffmann A. Communication in fragile X syndrome: patterns and implications for assessment and intervention. Frontiers in Psychology. 2022;13. 10.3389/fpsyg.2022.929379. 10.3389/fpsyg.2022.929379 PMC9817301 36619013

[30] Hall SS, Walter E, Sherman E, Hoeft F, Reiss AL. The neural basis of auditory temporal discrimination in girls with fragile X syndrome. J Neurodevelopmental Disorders. 2009;1(1):91–9. 10.1007/s11689-009-9007-x PMC2772079 19890439

[31] Rojas DC, Benkers TL, Rogers SJ, Teale PD, Reite ML, Hagerman RJ. Auditory evoked magnetic fields in adults with fragile X syndrome. NeuroReport. 2001;12(11):2573–6. 11496151 10.1097/00001756-200108080-00056

[32] Lawrence RJ, Wiggins IM, Hodgson JC, Hartley DEH. Evaluating cortical responses to speech in children: a functional near-infrared spectroscopy (fNIRS) study. Hear Res. 2021;401:108155. 33360183 10.1016/j.heares.2020.108155 PMC7937787

[33] Quaresima V, Bisconti S, Ferrari M. A brief review on the use of functional near-infrared spectroscopy (fNIRS) for language imaging studies in human newborns and adults. Brain Lang. 2012;121(2):79–89. 21507474 10.1016/j.bandl.2011.03.009

[34] Lloyd-Fox S, Blasi A, Pasco G, Gliga T, Jones EJH, Murphy DGM, Elwell CE, Charman T, Johnson MH, Team B. Cortical responses before 6 months of life associate with later autism. Eur J Neurosci. 2018;47(6):736–49. 29057543 10.1111/ejn.13757 PMC5900943

[35] Pecukonis M, Perdue KL, Wong J, Tager-Flusberg H, Nelson CA. Exploring the relation between brain response to speech at 6-months and language outcomes at 24-months in infants at high and low risk for autism spectrum disorder: a preliminary functional near-infrared spectroscopy study. Dev Cogn Neurosci. 2021;47:p100897. 10.1016/j.dcn.2020.100897 PMC7750322 33338817

[36] Li R, Bruno JL, Jordan T, Miller JG, Lee CH, Bartholomay KL, et al. Aberrant neural response during face processing in girls with fragile X syndrome: defining potential brain biomarkers for treatment studies. Biological Psychiatry: cognitive Neuroscience and Neuroimaging. 2023;8(3):311–19. 10.1016/j.bpsc.2021.09.003. 10.1016/j.bpsc.2021.09.003 PMC8964834 34555563

[37] Li R, Bruno JL, Lee CH, Bartholomay KL, Sundstrom J, Piccirilli A, et al. Aberrant brain network and eye gaze patterns during natural social interaction predict multi-domain social-cognitive behaviors in girls with fragile X syndrome. Mol Psychiatry. 2022;27(9):3768–76. 10.1038/s41380-022-01626-3. 10.1038/s41380-022-01626-3 PMC12135919 35595977

[38] Sparrow SS, Cicchetti DV, Balla DA. Vineland adaptive behavior scales: second edition (vineland II), the expanded interview form. Livonia, MN: Pearson Assessments; 2008.

[39] Mullen EM. Mullen scales of early learning. Circle Pines, MN: American Guidance Service Inc; 1995.

[40] Roid GH, Pomplun M. The Stanford-Binet Intelligence Scales, Fifth Edition, in Contemporary intellectual assessment: Theories, tests, and issues, 3rd ed. 2012, The Guilford Press: New York, NY, US. pp. 249–268.

[41] Bishop SL, Guthrie W, Coffing M, Lord C. Convergent validity of the Mullen scales of Early Learning and the differential ability scales in children with autism spectrum disorders. Am J Intellect Dev Disabil. 2011;116(5):331–43. 21905802 10.1352/1944-7558-116.5.331 PMC7398154

[42] Thurm A, Manwaring SS, Cardozo Jimenez C, Swineford L, Farmer C, Gallo R, Maeda M. Socioemotional and behavioral problems in toddlers with language delay. Infant Mental Health Journal: Infancy Early Child. 2018;39(5):569–80. 10.1002/imhj.21735 PMC6245647 30105861

[43] Oldfield RC. The assessment and analysis of handedness: the Edinburgh inventory. Neuropsychologia. 1971;9(1):97–113. 5146491 10.1016/0028-3932(71)90067-4

[44] Lord C, Rutter M, DiLavore PC, Risi S, Gotham K, Bishop S. Autism diagnostic observation schedule, second edition. 2012, Torrance, CA: Western Psychological Services.

[45] Wiggins LD, Barger B, Moody E, Soke G, Pandey J, Levy S. Brief report: the ADOS calibrated severity score best measures Autism Diagnostic Symptom Severity in Pre-school Children. J Autism Dev Disord. 2019;49(7):2999–3006. 28265795 10.1007/s10803-017-3072-x PMC5756129

[46] Wang Y, Wu M, Wu K, Liu H, Wu S, Zhang Z, Liu M, Wei C, Zhang Y-X, Liu Y. Differential auditory cortical development in left and right cochlear implanted children. Cereb Cortex. 2022;32(23):5438–54. 35165693 10.1093/cercor/bhac025

[47] Zhang YF, Lasfargues-Delannoy A, Berry I. Adaptation of stimulation duration to enhance auditory response in fNIRS block design. Hear Res. 2022;424:108593. 35964453 10.1016/j.heares.2022.108593

[48] Sroka MC, Vannest J, Maloney TC, Horowitz-Kraus T, Byars AW, Holland SK, Consortium CA. Relationship between receptive vocabulary and the neural substrates for story processing in preschoolers. Brain Imaging Behav. 2015;9(1):43–55. 25533780 10.1007/s11682-014-9342-8

[49] Schmithorst VJ, Holland SK, Plante E. Cognitive modules utilized for narrative comprehension in children: a functional magnetic resonance imaging study. NeuroImage. 2006;29(1):254–66. 16109491 10.1016/j.neuroimage.2005.07.020 PMC1357541

[50] Hickok G, Poeppel D. The cortical organization of speech processing. Nat Rev Neurosci. 2007;8(5):393–402. 17431404 10.1038/nrn2113

[51] Lindell AK, Hudry K. Atypicalities in cortical structure, handedness, and functional lateralization for Language in Autism Spectrum disorders. Neuropsychol Rev. 2013;23(3):257–70. 23649809 10.1007/s11065-013-9234-5

[52] Seery AM, Vogel-Farley V, Tager-Flusberg H, Nelson CA. Atypical lateralization of ERP response to native and non-native speech in infants at risk for autism spectrum disorder. Dev Cogn Neurosci. 2013;5:10–24. 23287023 10.1016/j.dcn.2012.11.007 PMC3676696

[53] Kraemer HC, Yesavage JA, Taylor JL, Kupfer D. How can we learn about developmental processes from cross-sectional studies, or can we? Am J Psychiatry. 2000;157(2):163–71. 10671382 10.1176/appi.ajp.157.2.163

[54] Croom K, Rumschlag JA, Erickson MA, Binder DK, Razak KA. Developmental delays in cortical auditory temporal processing in a mouse model of Fragile X syndrome. J Neurodevelopmental Disorders. 2023;15(1):23. 10.1186/s11689-023-09496-8 PMC10386252 37516865

[55] Steinbrink J, Villringer A, Kempf F, Haux D, Boden S, Obrig H. Illuminating the BOLD signal: combined fMRI–fNIRS studies. Magn Reson Imaging. 2006;24(4):495–505. 16677956 10.1016/j.mri.2005.12.034

[56] Cui X, Bray S, Bryant DM, Glover GH, Reiss AL. A quantitative comparison of NIRS and fMRI across multiple cognitive tasks. NeuroImage. 2011;54(4):2808–21. 21047559 10.1016/j.neuroimage.2010.10.069 PMC3021967

[57] Okamoto M, Dan H, Sakamoto K, Takeo K, Shimizu K, Kohno S, Oda I, Isobe S, Suzuki T, Kohyama K, Dan I. Three-dimensional probabilistic anatomical cranio-cerebral correlation via the international 10–20 system oriented for transcranial functional brain mapping. NeuroImage. 2004;21(1):99–111. 14741647 10.1016/j.neuroimage.2003.08.026

