# Supplementary material for: Specialization of the brain for language in children with Fragile X Syndrome: a functional Near Infrared Spectroscopy study
Source: J Neurodev Disord. 2024 Dec 19;16:69. doi: 10.1186/s11689-024-09582-5 (PMC11657644; doi:10.1186/s11689-024-09582-5)
Supplement: Supplementary file 1 — Supplementary Material 1 [file 11689_2024_9582_MOESM1_ESM.docx]

**Supplemental Information: Results without covariates**

*Activation for Speech and Non-speech in Controls*

Controls showed activation to speech in three left hemisphere channels (channel 2_1 t(13)=-3.18, p = .008; channel2_2 t(11)=-2.14, p = .045; channel 2_3 t(11)=-2.73, p = .013). Controls showed activation to speech in two right hemisphere channels as well (channel 5_6, t(14)=-2.1, p = .042, channel 7_6 t(14)=-3.78, p = .002). Controls showed activation to non-speech in two right hemisphere channels (channel 6_6 t(13)=-2.24, p = .035; channel 7_6 t(14)=-3.47, p = .0006). When looking across all 20 channels, the TDC group did not show an effect of side (left vs right) on activation to the speech stimuli (t(15) = .70,p = .49) or nonspeech stimuli (t(15)=-.67, p = .50).

Individuals with FXS, however, showed activation to speech in two left hemisphere channels (channel 2_1 t(20)=-3.06, p = .0065; channel 2_3 t(18)=-3.72, p = .0017) and in one right hemisphere channel (channel 7_6 t(19)=-3.03, p = .005). Individuals with FXS showed activation to non-speech sounds across three left hemisphere channels (channel 2_2 t(18)=-2.75, p = .014; channel 2_3 t(18)=-3.60, p = .0022; channel 3_3 t(14)=-2.41, p = .023). In the right hemisphere, individuals with FXS showed activation for non-speech sounds across three channels (channel 6_6 t(19)=-1.92, p = .06; channel 7_6 t(19)=-2.17, p = .037; channel 7_8 t(18)=-2.07, p = .05). Individuals with FXS did show greater activation in the left compared to right cortex for speech when looking across all 20 channels (t(22) = 2.43, p = .015) but did not show an effect of side on activation for nonspeech stimuli (t(23)=-.15, p = .88).

After correction for multiple comparisons, the only significant effects remaining included the TDC response to non-speech in channel 7_6 and the FXS response to speech in channel 2_3.

*Discrimination of speech and non-speech sounds*

We located channels where activation for the speech condition and for the non-speech condition varied significantly by determining relative differences in changes in Hb and HbO from baseline by condition (speech vs non-speech). Controls showed neural discrimination in the lateral most anterior channel in the left hemisphere (channel 2_1, between 10–20 coordinates T7 and FC7, t(13)=-2.66,p = .01, Cohen’s d = 1.5). Examination of waveforms shows greater activation for speech versus non-speech in TDC (Fig. 3). Individuals in the TDC group did not show differential discrimination of speech and nonspeech stimuli by hemisphere (left, right) across all 20 channels (t(15) = .95, p = .34) but did show greater differentiation of speech and nonspeech in the left versus right hemisphere at the channel level (for channels 2_2 versus 6_6, t(13) = 2.31, p = .024, Cohen’s d = 1.28).

Individuals with FXS did not show significant discrimination of speech vs non-speech sounds in any channel (e.g., channel 2_1, t(20=-1.35, p = .18, Cohen’s d = .6)). They did not show differential discrimination by hemisphere across all channels (t(23) = 1.735, p = .08, Cohen’s d = .72), and did not show hemispheric effects within channels (all p’s > .05).

Neither finding in the TDC group (discrimination of speech and non-speech at channel 2_1 nor hemispheric differences in differentiation at channels 2_2 and 6_6) remained significant after correction for multiple comparisons.

*Post Hoc*

Analyses at individual channels showed robust activity for the non-speech condition in FXS compared to controls, as seen in Fig. 2. The FXS group showed greater activation for the non-speech condition than TDC across the left and right auditory cortex combined (t(38) = 2.47,p = .014), which was also significant within the right hemisphere (t(38) = 1.97, p = .05), but not the left hemisphere (t(38) = 1.88, p = .06). This effect was only significant within one channel (channel 6_6 in the right hemisphere, t(34) = 1.99, p = .05). Differential processing of non-speech in FXS vs TDC across the auditory cortex did not remain significant after correction for multiple comparisons.

*Relation to language ability*

We investigated relation between significant findings above and language ability separately for the FXS and TDC group. Within the FXS group, neural discrimination of speech and nonspeech at channel 2_1 (where TDC group showed neural discrimination) did not predict scores of any language measures (for Verbal DQ, t(17)=-.52, p = .608; Vineland Receptive Language, t(20)=-1.27, p = .21; Vineland Expressive Language, t(20)=-.49, p = .62). Degree of neural lateralization for speech across all channels was not predicted by Verbal DQ (t(19) = 1.472, p = .141). However, Vineland Receptive and Expressive Language scores were associated with lateralization for speech (for VABS receptive, t(22) = 2.28, p = .02; for VABS expressive t(22) = 1.93, p = .053). Specifically, higher parent-report of receptive and expressive language was associated with greater leftward lateralization for speech stimuli. Increased response to non-speech stimuli across all 20 channels was not significantly correlated with Verbal DQ (t(20) = 1.87, p = .061). Response to non-speech stimuli across all 20 channels was not associated with Vineland Receptive or Expressive language scores (t(23) = .57, p = .57; t(23)=-1.34, p = .18). The relation between Vineland language scores and leftward lateralization of speech stimuli did not remain significant after correction for multiple comparisons (for RL, Cohen’s d = .97; for EL, d = .82).

In the TDC group, we investigated the relation between the above findings and scores on the Vineland receptive and expressive subscales, but did not investigate relation to Developmental Quotient due to missing data (TDC n with DQ data = 5). For the TDC group, neural discrimination of speech and nonspeech stimuli at channel 2_1 was not associated with Vineland receptive language (t(11)=-.69, p = .49), but was associated with Vineland expressive language (t(11)=-2.10, p = .04). Lateralization for the speech condition was not associated with Vineland receptive language (t(13) = 1.09, p = .28), but was associated with Vineland expressive language (t(13) = 2.03, p = .04). Specifically, higher parent report of expressive language use was associated with increased discrimination of speech and nonspeech and with increased leftward lateralization for speech. Response to the nonspeech stimulus across the brain was not associated with either expressive or receptive language on the Vineland for the TDC group (receptive t(13) = .54, p = .59; expressive t(13) = .57, p = .57).
